# HIV transmission and associated factors under the scale-up of HIV antiretroviral therapy: a population-based longitudinal molecular network study

**DOI:** 10.1186/s12985-023-02246-1

**Published:** 2023-12-04

**Authors:** Yi Chen, Zhiqiang Cao, Jianjun Li, Jin Chen, Qiuying Zhu, Shujia Liang, Guanghua Lan, Hui Xing, Lingjie Liao, Yi Feng, Yiming Shao, Yuhua Ruan, Huanhuan Chen

**Affiliations:** 1 The People’s Hospital of Guangxi Zhuang Autonomous Region and Guangxi Academy of Medical Sciences, Nanning, 530021 China; 2 State Key Laboratory of Infectious Disease Prevention and Control (SKLID), Collaborative Innovation Center for Diagnosis and Treatment of Infectious Diseases, National Center for AIDS/STD Control and Prevention (NCAIDS), Chinese Center for Disease Control and Prevention (China CDC), Beijing, 102206 China; 3 Guangxi Key Laboratory of Major Infectious Disease Prevention Control and Biosafety Emergency Response, Guangxi Center for Disease Control and Prevention, Nanning, 530028 China

**Keywords:** HIV/AIDS, Longitudinal molecular network, Antiretroviral therapy, Generalized estimating equation

## Abstract

**Objectives:**

To evaluate the prevention efficacy of scaling up HIV/AIDS antiretroviral therapy (ART) on HIV transmission at the population level and determine associated factors of HIV secondary transmission.

**Methods:**

We used HIV longitudinal molecular networks to assess the genetic linkage between baseline and newly diagnosed cases. A generalized estimating equation was applied to determine the associations between demographic, clinical characteristics and HIV transmission.

**Results:**

Patients on ART had a 32% lower risk of HIV transmission than those not on ART. A 36% reduction in risk was also seen if ART-patients maintained their HIV viral load lower than 50 copies/mL. A 71% lower risk occurred when patients sustained ART for at least 3 years and kept HIV viral load less than 50 copies/mL. Patients who discontinued ART had a similar HIV transmission risk as those not on ART. Patients who were older, male, non-Han, not single, retired, infected via a heterosexual route of transmission and those who possessed higher CD4 counts had a higher risk of HIV transmission. HIV-1 subtype of CRF01_AE was less transmissible than other subtypes.

**Conclusions:**

The efficacy of ART in a real-world setting was supported by this longitudinal molecular network study. Promoting adherence to ART is crucial to reduce HIV transmission.

**Supplementary Information:**

The online version contains supplementary material available at 10.1186/s12985-023-02246-1.

## Introduction

In 2011 the HIV Prevention Trials Network 052 (HPTN 052) reported that HIV transmission between sero-discordant heterosexual couples could be reduced by 96% when the HIV-infected partner received early antiretroviral therapy (ART) [1–3]. In addition to this well-designed randomized controlled trial, many observational cohort studies based in real-world settings of national and provincial or regional levels in China during 2012–2018 found a strong effect of ART in reducing the risk of HIV transmission in sero-discordant couples [4–6]. Based on the evidence from such studies, the Joint United Nations Programme on HIV/AIDS (UNAIDS) promoted the “95 − 95 − 95” targets in 2014 aiming to diagnose 95% of all people living with HIV (PLHIV), provide ART for 95% of those diagnosed, and expect that 95% of all people receiving antiretroviral therapy will have viral suppression by 2030 [7]. China implemented the “treat all” policy in late 2016 [8, 9], which further scaled up the ART coverage in the communities. HPTN 071 (PopART) provided evidence in 2019 that universal testing and treatment can reduce the incidence of HIV at the population level in real-world settings [10]. However, few studies have described community-based HIV transmission risks under the scale up of ART in real-world settings. Additionally, little is known about the characteristics of secondary HIV transmission in the communities after the “treat all” policy was adopted. Prospective cohort studies of HIV sero-incidence are time-consuming, and costly in terms of human, material and financial resources. Loss to follow-up is also difficult to control. With the goal of ensuring that all people living with HIV (PLHIV) across all demographics and geographic settings achieving the three “95” targets by 2030, an evaluation of HIV transmission risks and related characteristics in communities under the scale up of ART is now an urgent need.

With the development and application of genetic sequencing techniques, more HIV sequences can now be used in HIV prevention studies. Under certain genetic distance thresholds, an HIV molecular network can be constructed using HIV sequences achieved from groups of people infected with a similar virus strain [11]. The HIV molecular network can be integrated with epidemiological characteristics and ART status of patients. This unique advantage has also been demonstrated in evaluation of HIV transmission. Two US studies found that timely monitoring of the growth of a molecular network can help to identify the future transmission cluster and detect recent outbreaks [12, 13]. US molecular analysis studies found that young men who have sex with men (MSM) were the high risk group in driving the local HIV transmission [14, 15]. US and China researchers made use of molecular networks to distinguish non-disclosed MSM among self-reported heterosexual men [16, 17]. Molecular transmission networks have also been employed to identify the transmission route between different subgroups, which can contribute to more precise interventions [18].

In addition, molecular networks have been applied in the evaluation of HIV prevention and control strategies. Our previous study established a longitudinal molecular network method to evaluate the effects of HIV treatment in reducing HIV transmission in a real-world setting [19]. However, a crucial limitation in that study was that it did not consider the effect of patients’ demographic and clinical characteristics on the efficacy of ART towards HIV prevention. The generalized estimating equation (GEE) method can adjust for possible unmeasured correlations between observations from repeated measurements [20]. We will employ a GEE framework to evaluate the prevention efficacy of scaling up ART on HIV transmission at the population level and determine associated factors of HIV secondary transmission.

## Materials and methods

### Study setting

The study was conducted in Qinzhou, Guangxi Zhuang Autonomous Region (herein “Guangxi”). Guangxi accounts for more than 10% of the total HIV incidence in China while the population accounts for only 4% of the national population [21]. Qinzhou is a prefecture located in southern Guangxi where the number of HIV diagnosed cases ranks among the top three in the region.

### Study design and participants

**Fig. 1:**
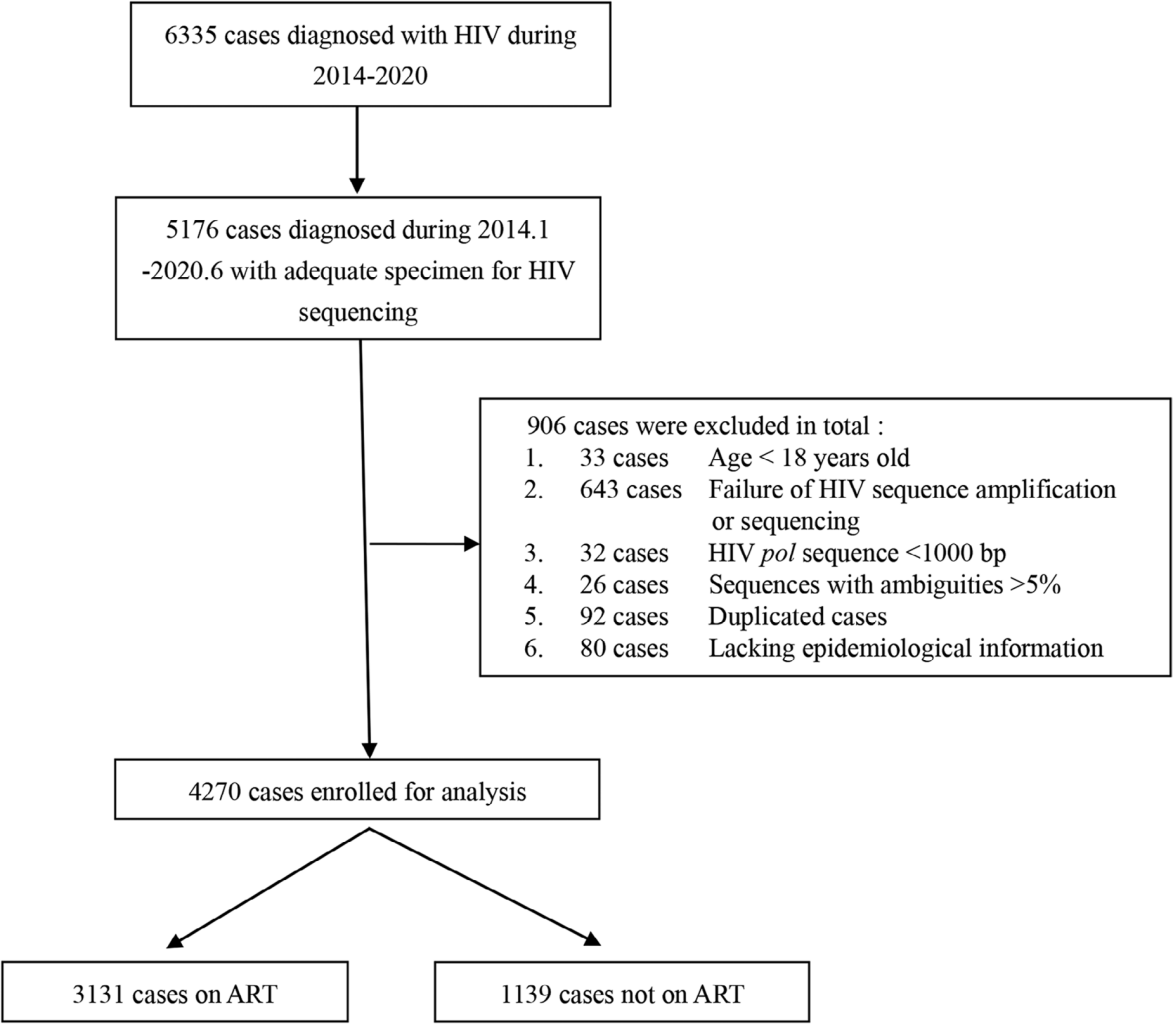
Flowchart of study samples selection

This is a prospective longitudinal molecular network study. Figure 1 shows the details of samples selection. The study population covered the HIV/AIDS cases diagnosed between January 2014 and June 2020. The excluded criteria included (1) Age < 18 years, (2) Failure of HIV amplification or sequencing, (3) HIV *pol* sequence < 1000 bp, (4) Sequences with ambiguities > 5%, (5) Duplicated cases, (6) Lacking epidemiological information. To determine the transmission occurrence between baseline cases and newly diagnosed cases, we used the following definitions. For cases newly diagnosed in 2017, their corresponding baseline cases were those diagnosed between 2014 and 2016. For cases newly diagnosed in 2018, the corresponding baseline cases were those diagnosed between 2014 and 2017. For cases newly diagnosed in 2019, the corresponding baseline cases were those diagnosed between 2014 and 2018. For cases newly diagnosed in 2020, the corresponding baseline cases were those diagnosed between 2014 and 2019. All eligible study subjects provided written informed consent. The study was approved by the institutional review board of National Center for AIDS/STD Control and Prevention, Chinese Center for Disease Control and Prevention, China (X140617334).

### Demographic and clinical data collection

The demographic and clinical data for the participants were collected from Guangxi Qinzhou HIV/AIDS Comprehensive Prevention and Control Information System. The demographic data included age, gender, ethnicity, education, marital status, occupation, and route of HIV transmission. Clinical data included date of HIV diagnosis, CD4 + count at diagnosis, year of ART initiation, viral load level, whether the subject dropped out of ART, duration of ART and survival status. As for HIV patients newly diagnosed, follow-up visits were conducted every 3 months before initiation of ART. Follow-up visits were conducted at 0.5, 1, 2 and 3 months after initiation of ART and then every 3 months. The national guidelines require that one or more viral load tests be performed every year after ART initiation as routine viral load monitoring for HIV/AIDS cases on ART. We used the updated ART and viral load data for the study samples. When looking at the linkages between newly diagnosed cases in 2017 and baseline cases in 2014–2016, the newest ART status and HIV viral load data on December 31, 2016 for the baseline cases diagnosed in 2014–2016 was used; when considering the linkages between newly diagnosed cases in 2018 and corresponding baseline cases in 2014–2017, the updated ART status and HIV viral load data on December 31, 2017 for the second baseline cases diagnosed in 2014–2017 was used, and so on. A “drop out” was defined as withdrawal or loss to follow-up for more than 90 days [8].

### HIV-1 RNA extraction, amplification and sequencing

Whole blood samples were collected from a pool of registered HIV/AIDS patients in Qinzhou, Guangxi. Plasma was isolated from the blood specimens and sent to the central Laboratory under a cold chain. Ribonucleic acid (RNA) was extracted from plasma (200mL) based on the QIAsymphony platform. The pol fragment (protease 1–99 amino acids and part of reverse transcriptase 1–250 amino acids, HXB2 positions 2253–3312) was amplified and sequenced using an inhouse polymerase chain reaction protocol according to previously published methods [22]. Quality control was performed simultaneously, in order to achieve a more precise molecular transmission network, we eliminated 32 sequences that were less than 1000 nucleotides in length and excluded 26 cases whose sequences contained ambiguities > 5% [23].

### HIV molecular network construction

An HIV molecular cluster is a group of persons diagnosed with HIV who have genetically similar HIV strains [11]. The sequences were spliced by BioEdit (Ibis Biosciences, Carlsbad, CA, United States; version 7.0.9.0) and aligned separately by the HIV align tool to obtain the final sequences used for analysis. MEGA version 10.0 was used to identify the HIV-1 subtypes, RaxmlGUI version 2.0.0 was used to construct the phylogenetic tree. Before constructing the molecular transmission network, the pairwise Tamura-Nei (TN93) genetic distances of the paired sequences were calculated using Hyphy version 2.2.5. Finally, the HIV molecular transmission network was visualized using Cytoscape version 3.2.1.

**Fig. 2:**
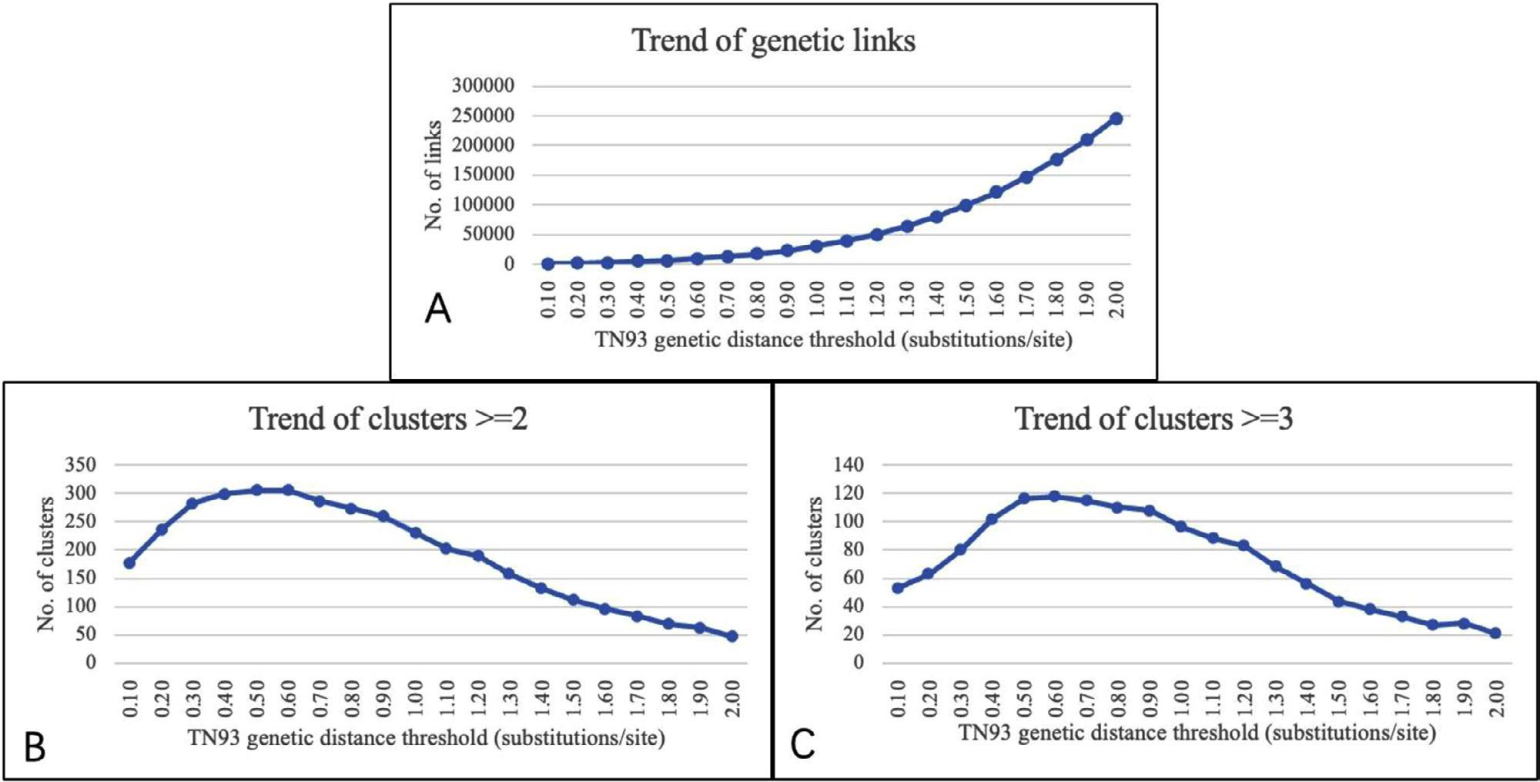
Trend of links and clusters in the genetic network under different genetic distance thresholds. Legend: The genetic distance threshold is based on Tamura and Nei 93(TN93) model; All of the sample sequences were based in Qinzhou, Guangxi and diagnosed between 2014 and 2020

Figure 2 illustrates the number of genetic links and molecular clusters across different genetic distance thresholds (0.1–2.0%). All of the enrolled HIV sequences were used to determine the optimal genetic distance threshold. It can be seen from the figure that the optimal genetic distance threshold is 0.0050 substitutions/site with maximum number of clusters identified [23]. When the genetic distance threshold was less than 0.0050 substitutions/site, the number of nodes and clusters (nodes ≥ 2 or ≥ 3) increased as genetic distance expanded. When the genetic distance threshold reached 0.0050 substitutions/site, the number of clusters (nodes ≥ 2 or ≥ 3) achieved the maximum. When the genetic distance threshold exceeded 0.0050 substitutions/site, the number of clusters (nodes ≥ 2 or ≥ 3) decreased. This indicated that the merged speed of clusters (nodes ≥ 2 or ≥ 3) was higher than that of such clusters emerging. A genetic distance threshold of 0.0050 substitutions/site corresponds to approximately a maximum of 2–3 years of viral evolution separating these strains, which may correspond to time since a common transmission event [11]. In this study, we chose 0.0050 substitutions/site to construct a molecular transmission network in order to find more clusters with more recent transmissions. This network was used for subsequent analysis and visualized with singletons omitted.

### Calculation of genetic linkages between baseline cases and newly diagnosed cases with HIV

Regarding calculation of genetic linkages between baseline cases and newly diagnosed cases with HIV, we developed a method based on the network linkage and applied it in a previous study [19]. First, patients diagnosed with HIV during 2014–2016 were defined as first baseline network and the corresponding newly diagnosed cases were those diagnosed in the following year (2017). The genetic linkages between newly diagnosed patients with HIV in 2017 and baseline cases diagnosed in 2014–2016 were thus calculated. We then used the molecular transmission network containing cases diagnosed during 2014–2017 as a second baseline network, calculated the linkage between them and cases newly diagnosed in 2018. Similarly, the linkages between cases newly diagnosed in 2019 and 2020 and their corresponding baseline networks were calculated. The number of genetic linkages was equal to the number of newly diagnosed patients with HIV linked to those at the corresponding baseline molecular network. The genetic linkages among samples diagnosed in 2014–2016 were not taken into account since they were used as a basic baseline group sample for our study.

### Statistical analysis

After constructing the HIV molecular transmission network based on the optimal genetic distance threshold, the distribution of study samples diagnosed in 2014–2016, 2017, 2018, 2019 and 2020 with epidemiological characteristics and clinical information were described.

The outcome variable in the study is an ordinal variable, the levels included whether a case in baseline molecular transmission network had a genetic linkage with (1) zero case in the corresponding newly diagnosed cases molecular transmission network, (2) one case in corresponding newly diagnosed cases molecular transmission network, and (3) at least two cases in the corresponding newly diagnosed cases molecular transmission network. GEE models are an extension of generalized linear models (GLM). This framework can handle data with a non-normally distributed dependent variable and account for the correlation among the multiple observations that arise from a single subject and can largely estimate the correlations within observations without having to accurately specify the form of the correlation structure [24]. In this case, it is better than GLM in predicting the associations between patient’s demographic characteristics, clinical information and their HIV transmission based on longitudinal molecular transmission networks between baseline cases and those newly diagnosed cases. Furthermore, we developed three models based on patients ART status and HIV viral load level to analyze the HIV transmission effects. In Model 1, the patients were divided into three levels: “not on ART”, “on ART” and “Dropped out”. In Model 2, the patients were separated into five groups based on their HIV viral load: (1) not on ART, (2) less than 50 copies/mL, (3) viral load between 50 and 999 copies/mL, (4) viral load at least 1000 copies/mL, and (5) unknown viral load. In Model 3, the patients were divided into two groups, the first group consists of patients on ART for at least 3 years and having a viral load less than 50 copies/mL, while the remaining patients belong to the second group. Age, gender, ethnicity, education, marital status, occupation, route of HIV transmission, year of HIV diagnosis, HIV-1 subtype and CD4 + count at diagnosis were included as covariates in all GEE models. All statistical analyses were performed using SAS version 9.4.

## Results

### Characteristics of study population

Table 1 shows detailed information on demographic characteristics, HIV epidemic and clinical characteristics of study samples diagnosed from 2014–2016 to 2020 in Qinzhou. The total number of cases was 4270. In addition to the basic baseline cases diagnosed in 2014–2016, the number of newly diagnosed cases ranged from 691 in 2019 to 201 in 2020. Cases diagnosed in 2014–2016 were analyzed as a group sample. The purpose of separating samples into 2014–2016, 2017, 2018, 2019 and 2020 is to emphasize the time point of implementation of “treat all” policy at the end of 2016.

**Table 1:** Characteristics of newly diagnosed HIV/AIDS cases between 2014–2016 and 2020 in Qinzhou, Guangxi

Variable	Total	Year of diagnosis				
		2014–2016	2017	2018	2019	2020
Total	4270	2176	595	607	691	201
Age (years)						
18 ~ 29	413 (9.7)	240(11.0)	62 (10.4)	53 (8.7)	44 (6.4)	14 (7.0)
30 ~ 49	1701 (39.8)	1014(46.6)	220 (37.0)	192 (31.6)	228 (33)	47 (23.4)
50 ~ 69	1774 (41.5)	745(34.2)	260 (43.7)	304 (50.1)	352 (50.9)	113 (56.2)
≥ 70	382 (8.9)	177(8.1)	53 (8.9)	58 (9.6)	67 (9.7)	27 (13.4)
Gender						
Male	3191 (74.7)	1654(76.0)	441 (74.1)	453 (74.6)	489 (70.8)	154 (76.6)
Female	1079 (25.3)	522(24.0)	154 (25.9)	154 (25.4)	202 (29.2)	47 (23.4)
Ethnicity						
Han	3872 (90.7)	1987(91.3)	540 (90.8)	542 (89.3)	614 (88.9)	189 (94.0)
Zhuang	352 (8.2)	160(7.4)	54 (9.1)	61 (10.0)	66 (9.6)	11 (5.5)
Other	46 (1.1)	29(1.3)	1 (0.1)	4 (0.7)	11 (1.6)	1 (0.5)
Education						
Illiteracy	190 (4.4)	88(4.0)	25 (4.2)	26 (4.3)	43 (6.2)	8 (4.0)
Elementary school	2161 (50.6)	1073(49.3)	309 (51.9)	302 (49.8)	363 (52.5)	114 (56.7)
Secondary school	1560 (36.5)	832(38.2)	206 (34.6)	228 (37.6)	230 (33.3)	64 (31.8)
High school or above	359 (8.4)	183(8.4)	55 (9.2)	51 (8.4)	55 (8.0)	15 (7.5)
Marital status						
Single	996 (23.3)	576(26.5)	122 (20.5)	120 (19.7)	140 (20.3)	38 (18.9)
Married	2481 (58.1)	1227(56.4)	373 (62.7)	358 (59.0)	405 (58.6)	118 (58.7)
Divorced/Widowed	793 (18.6)	373(17.1)	100 (16.8)	129 (21.3)	146 (21.1)	45 (22.4)
Occupation						
Farmer	3234 (75.7)	1579(72.6)	458 (77.0)	487 (80.2)	556 (80.5)	154 (76.6)
Housekeeping	557 (13.0)	307(14.1)	71 (11.9)	68 (11.2)	75 (10.9)	36 (17.9)
Retired	83 (1.9)	45(2.0)	15 (2.5)	10 (1.6)	12 (1.7)	1 (0.5)
Other	396 (9.3)	245(11.3)	51 (8.6)	42 (6.9)	48 (6.9)	10 (5.0)
Transmission route						
Injecting drug use	393 (9.2)	330(15.2)	17 (2.9)	29 (4.8)	17 (2.5)	0 (0.0)
Heterosexual	3795 (88.9)	1814(83.4)	560 (94.1)	565 (93.1)	660 (95.5)	196 (97.5)
Homosexual	82 (1.9)	32(1.5)	18 (3.0)	13 (2.1)	14 (2.0)	5 (2.5)
HIV-1 subtype						
CRF01_AE	2350 (55.0)	1286(59.1)	348 (58.5)	307 (50.6)	326 (47.2)	83 (41.3)
Cluster 1	474 (11.1)	264(12.1)	73 (12.3)	53 (8.7)	66 (9.6)	18 (9.0)
Cluster 2	1687 (39.5)	903(41.5)	241 (40.5)	229 (37.7)	250 (36.2)	64 (31.8)
Other cluster	189 (4.4)	119(5.5)	34 (5.7)	25 (4.1)	10 (1.4)	1 (0.5)
CRF07_BC	395 (9.3)	146(6.7)	72 (12.1)	53 (8.7)	85 (12.3)	39 (19.4)
CRF08_BC	1275 (29.9)	649(29.8)	147 (24.7)	196 (32.3)	224 (32.4)	59 (29.4)
Other subtype#	250 (5.9)	95(4.4)	28 (4.7)	51 (8.4)	56 (8.1)	20 (10.0)
CD4 + counts at diagnosis (cells/m^3^)						
< 200	2189 (51.3)	1106(50.8)	287 (48.2)	343 (56.5)	345 (49.9)	108 (53.7)
200–349	984 (23.0)	441(20.3)	164 (27.6)	139 (22.9)	185 (26.8)	55 (27.4)
350–499	622 (14.6)	335(15.4)	84 (14.1)	84 (13.8)	97 (14.0)	22 (10.9)
≥ 500	401 (9.4)	253(11.6)	53 (8.9)	34 (5.6)	49 (7.1)	12 (6.0)
Missing	74 (1.7)	41(1.9)	7 (1.2)	7 (1.2)	15 (2.2)	4 (2.0)

# Other subtypes included CRF55_01B, B/B’ and other rare CRF and URF.

Most cases were aged between 50 and 69 years, similarly for cases diagnosed from 2017 to 2020. In the period of 2014–2016, the majority of individuals fell within the age range of 30 to 49 years. Overall, males constituted three-quarters of the cases, with percentages varying from 70.8 to 76.6% across each time point. More than 90% of all cases belonged to Han ethnicity. Approximately half had completed elementary school, with percentages ranging from 49.3 to 56.7% at each time point. Nearly 60.0% of cases were married (ranging from 56.4 to 62.7% at each time point) and identified as farmers (ranging from 72.6 to 80.5% at each time point). Almost 90.0% of HIV infections occurred through heterosexual transmission, and this percentage increased from 83.4% in 2014–2016 to 97.5% in 2020.The predominant HIV-1 subtype detected was CRF01_AE, accounting for 55.0% overall and ranging from 41.3 to 59.1% at each time point. CD4 + counts at diagnosis for over 50% cases was less than 200 cells/mm^3^, ranging from 48.2 to 56.5% across each time point.

### Molecular network linkage between baseline networks and newly diagnosed cases

Baseline molecular networks were constructed from 2176, 2771, 3378, and 4069 samples diagnosed in 2014–2016, 2014–2017, 2014–2018 and 2014–2019, respectively, of which 326 (15.0%), 422 (15.3%), 445 (13.2%), 311 (7.6%) cases were linked with at least one case newly diagnosed in 2017, 2018, 2019 and 2020 respectively.

### Association of factors with HIV transmission

#### Associations between patients’ demographic and clinical characteristics and HIV transmission

Overall, 12,394 subjects, possibly repeated, were observed in the molecular networks throughout 2014–2019, which were included in the GEE model for analysis. Among these, 762 (6.1%), 1504 (12.1%), and 742 (6.0%) were linked with one, at least one and at least two HIV cases newly diagnosed in the following years, respectively.

**Fig. 3:**
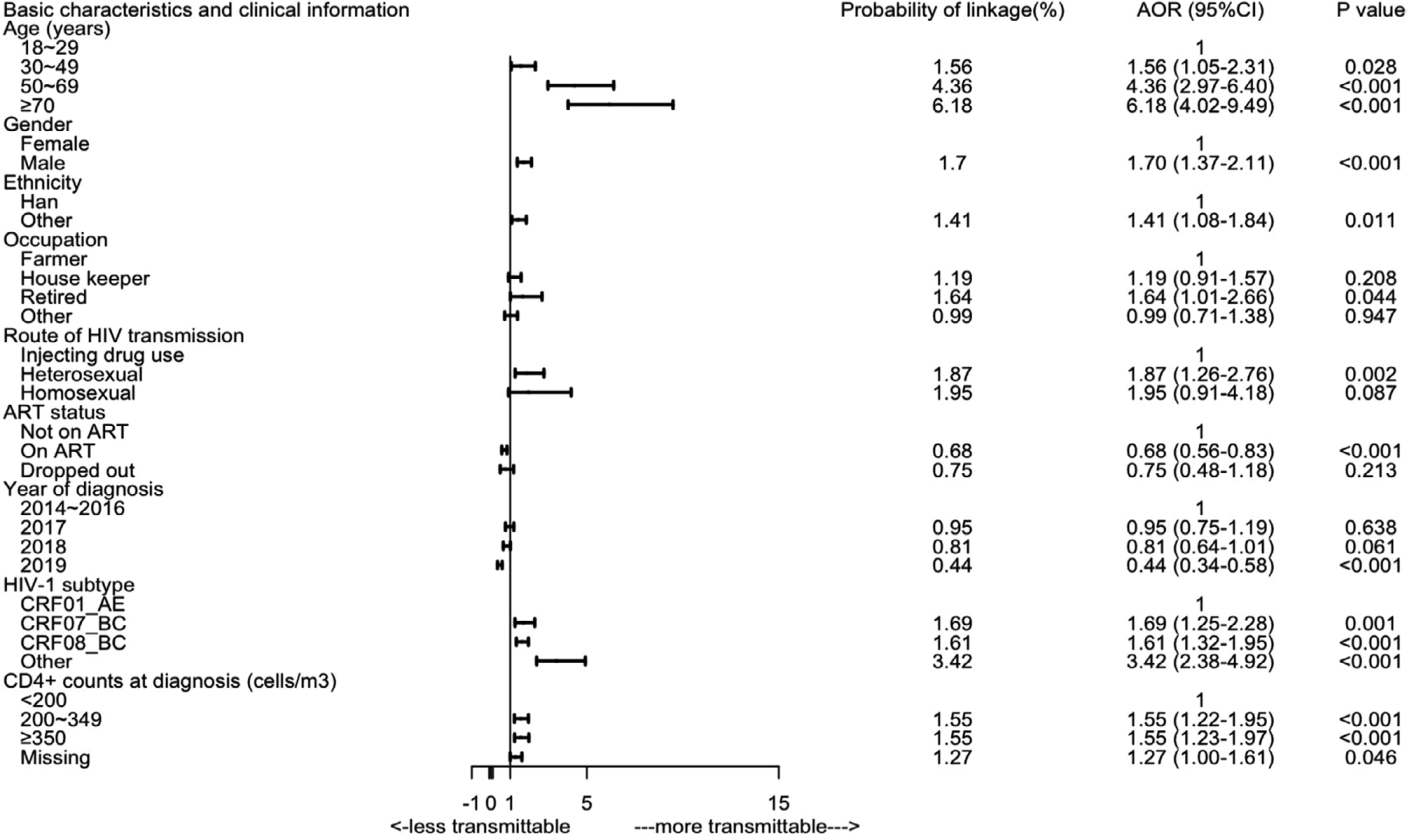
Adjusted odds ratios for HIV transmission by basic characteristics and clinical information in Qinzhou. *Note*: ART denotes Antiretroviral therapy

Table 2; Fig. 3 present the associations between demographic and clinical characteristics and HIV transmission. Compared to 18 ~ 29 age group, older age at baseline was associated with a higher risk of HIV transmission. Men had a higher risk than women (AOR:1.70,95% CI:1.37–2.11). Minority ethnic groups were more likely to be linked with newly diagnosed HIV cases than those of Han ethnicity (AOR:1.41, 95% CI:1.08–1.84), as were retirees compared with farmers (AOR:1.64, 95% CI:1.01–2.66) and those infected via heterosexual intercourse (AOR:1.87, 95% CI:1.26–2.76). Those on ART had a significantly lower HIV transmission risk compared to those not on ART (AOR:0.68, 95% CI:0.56–0.83). Cases who dropped out from ART had a similar probability of HIV transmission as those not on ART (AOR:0.75, 95%CI:0.48–1.18). Those diagnosed in 2019 had significantly fewer linkages with newly diagnosed HIV cases compared to baseline cases diagnosed in 2014–2016(AOR:0.44,95%CI:0.34–0.58). Compared to HIV-1 subtype of CRF01_AE, CRF07_BC(AOR:1.69,95%CI:1.25–2.28), CRF08_BC(AOR:1.61,95%CI:1.32–1.95) and other subtypes (AOR:3.42,95%CI:2.38–4.92) were more transmissible. Those with CD4 + counts at diagnosis not less than 200(200–349 and ≥ 350) had a higher risk of HIV transmission compared with those whose CD4 + counts were lower than 200.

**Table 2:** HIV molecular linkage between baseline cases during 2014–2019 and newly diagnosed cases during 2017–2020 in Qinzhou, respectively

Variable	Baseline cases	No. of linkages between newly diagnosed cases and baseline cases n (%)				Crude OR^¶^ (95% CI)	*P-value*	Adjusted OR (95% CI)	*P-value*
		0	1	≥ 2	≥ 1				
Total	12,394	10,890(87.9)	762 (6.1)	742 (6.0)	1504 (12.1)				
Age (years)									
18 ~ 29	1296	1233(95.1)	41 (3.2)	22 (1.7)	63 (4.9)	1.00		1.00	
30 ~ 49	5328	4957(93.0)	218 (4.1)	153 (2.9)	371 (7.0)	1.47 (1.01–2.13)	0.043	1.56 (1.05–2.31)	0.028
50 ~ 69	4720	3909(82.8)	386 (8.2)	425 (9.0)	811 (17.2)	4.12 (2.88–5.89)	< 0.001	4.36 (2.97–6.40)	< 0.001
≥ 70	1050	791(75.3)	117 (11.1)	142 (13.5)	259 (24.7)	6.52 (4.38–9.71)	< 0.001	6.18 (4.02–9.49)	< 0.001
Gender									
Female	3060	2791(91.2)	141 (4.6)	128 (4.2)	269 (8.8)	1.00		1.00	
Male	9334	8099(86.8)	621 (6.7)	614 (6.6)	1235 (13.2)	1.58 (1.29–1.95)	< 0.001	1.70 (1.37–2.11)	< 0.001
Ethnicity									
Han	11,266	9938(88.2)	678 (6.0)	650 (5.8)	1328 (11.8)	1.00		1.00	
Other	1128	952(84.4)	84 (7.4)	92 (8.2)	176 (15.6)	1.39 (1.08–1.79)	0.010	1.41 (1.08–1.84)	0.011
Education									
Secondary school or above	6700	5785(86.3)	468 (7.0)	447 (6.7)	915 (13.7)	1.00			
Elementary school or below	5694	5105(89.7)	294 (5.2)	295 (5.2)	589 (10.3)	0.73 (0.62–0.86)	< 0.001		
Marital status									
Single	3050	2791(91.5)	151 (5.0)	108 (3.5)	259 (8.5)	1.00			
Married	7148	6205(86.8)	460 (6.4)	483 (6.8)	943 (13.2)	1.66 (1.34–2.04)	< 0.001		
Divorced/Widowed	2196	1894(86.2)	151 (6.9)	151 (6.9)	302 (13.8)	1.73 (1.35–2.23)	< 0.001		
Occupation									
Farmer	9220	8066(87.5)	587 (6.4)	567 (6.1)	1154 (12.5)	1.00		1.00	
House keeper	1652	1476(89.3)	98 (5.9)	78 (4.7)	176 (10.7)	0.83 (0.64–1.06)	0.141	1.19 (0.91–1.57)	0.208
Retired	257	184(71.6)	24 (9.3)	49 (19.1)	73 (28.4)	2.97 (1.87–4.70)	< 0.001	1.64 (1.01–2.66)	0.044
Other	1265	1164(92)	53 (4.2)	48 (3.8)	101 (8.0)	0.61 (0.45–0.82)	0.001	0.99 (0.71–1.38)	0.947
Route of HIV transmission									
Injecting drug use	1446	1369(94.7)	52 (3.6)	25 (1.7)	77 (5.3)	1.00		1.00	
Heterosexual	10,726	9315(86.8)	699 (6.5)	712 (6.6)	1411 (13.2)	2.73 (1.94–3.82)	< 0.001	1.87 (1.26–2.76)	0.002
Homosexual	222	206(92.8)	11 (5.0)	5 (2.3)	16 (7.2)	1.37 (0.69–2.74)	0.372	1.95 (0.91–4.18)	0.087
On ART*									
No	3838	3277(85.4)	298 (7.8)	263 (6.9)	561 (14.6)	1.00		1.00	
Yes	7955	7073(88.9)	442 (5.6)	440 (5.5)	882 (11.1)	0.76 (0.64–0.89)	< 0.001	0.68 (0.56–0.83)	< 0.001
Dropped out	601	540(89.9)	22 (3.7)	39 (6.5)	61 (10.1)	0.79 (0.51–1.23)	0.298	0.75 (0.48–1.18)	0.213
Year of diagnosis									
2014 ~ 2016	8704	7711(88.6)	508 (5.8)	485 (5.6)	993 (11.4)	1.00		1.00	
2017	1785	1530(85.7)	128 (7.2)	127 (7.1)	255 (14.3)	1.30 (1.04–1.61)	0.020	0.95 (0.75–1.19)	0.638
2018	1214	1034(85.2)	81 (6.7)	99 (8.2)	180 (14.8)	1.37 (1.10–1.70)	0.004	0.81 (0.64–1.01)	0.061
2019	691	615(89.0)	45 (6.5)	31 (4.5)	76 (11.0)	0.94 (0.73–1.22)	0.666	0.44 (0.34–0.58)	< 0.001
HIV-1 subtype									
CRF01_AE	7128	6391(89.7)	419 (5.9)	318 (4.5)	737 (10.3)	1.00		1.00	
CRF07_BC	991	818(82.5)	74 (7.5)	99 (10.0)	173 (17.5)	1.87 (1.42–2.47)	< 0.001	1.69 (1.25–2.28)	0.001
CRF08_BC	3653	3191(87.4)	230 (6.3)	232 (6.4)	462 (12.6)	1.27 (1.06–1.52)	0.009	1.61 (1.32–1.95)	< 0.001
Other^#^	622	490(78.8)	39 (6.3)	93 (15.0)	132 (21.2)	2.54 (1.80–3.58)	< 0.001	3.42 (2.38–4.92)	< 0.001
CD4 + counts at diagnosis (cells/m^3^)									
< 200	5040	4508(89.5)	288 (5.7)	244 (4.8)	532 (10.5)	1.00		1.00	
200 ~ 349	2167	1838(84.8)	156 (7.2)	173 (8.0)	329 (15.2)	1.54 (1.24–1.91)	< 0.001	1.55 (1.22–1.95)	< 0.001
≥ 350	2722	2364(86.8)	168 (6.2)	190 (7.0)	358 (13.2)	1.30 (1.05–1.61)	0.017	1.55 (1.23–1.97)	< 0.001
Missing	2465	2180(88.4)	150 (6.1)	135 (5.5)	285 (11.6)	1.11 (0.89–1.39)	0.354	1.27 (1.00-1.61)	0.046

¶ OR: Odds ratio

* ART: antiretroviral therapy

# Other subtypes included CRF55_01B, B/B’ and other rare CRF and URF.

Covariates of the adjusted model included: age, gender, ethnicity, education, marital status, occupation, route of transmission, year of diagnosis, subtype, CD4 + counts at diagnosis

#### 2) Associations between stratified participants’ ART status, HIV viral load level and HIV transmission

**Fig. 4:**
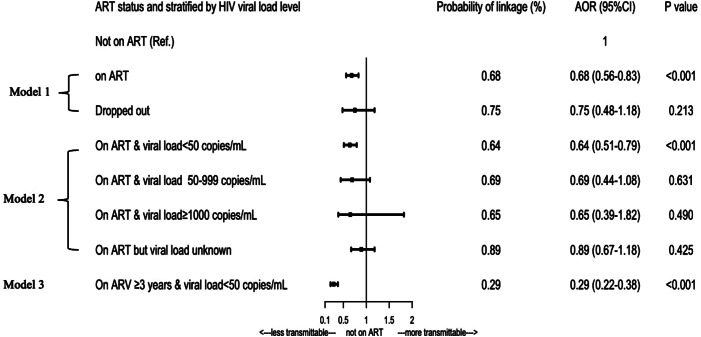
Adjusted odds ratios for HIV transmission by ART and viral load in Qinzhou. *Note*: ART denotes Antiretroviral therapy

Figure 4 demonstrates the associations between ART status, HIV viral load level and HIV transmission with further details provided in Table 3. In Model 1, it can be seen that comparing with not on ART, cases on ART was associated with less HIV transmission (AOR:0.68, 95% CI:0.56–0.83). In Model 2, cases having a viral load less than 50 copies/mL had a lower HIV transmission (AOR:0.64, 95% CI:0.51–0.79) comparing those not on ART. Those with a viral load at 50–999 copies/mL (AOR:0.69,95%CI:0.44–1.08) and not less than 1000 copies/mL (AOR:0.65, 95%CI:0.39–1.82) had a similar risk of HIV transmission as those not on ART, as well as cases with unknown viral loads (AOR:0.89, 95% CI:0.67–1.18). In Model 3, cases with sustained ART not less than three years and keeping HIV viral load level lower than 50 copies/mL had robust predictive capability in reductive HIV transmission (AOR:0.29,95%CI:0.22–0.38) than those not on ART.

**Table 3:** HIV transmission of molecular linkages between baseline HIV/AIDS cases during 2014–2019 and newly diagnosed cases during 2017–2020 in Qinzhou, Guangxi, stratified by treatment and HIV viral load level

Variable	Baseline cases	Linkages between newly diagnosed cases and baseline cases n (%)				Crude OR^¶^ (95% CI)	*P-value*	Adjusted OR (95% CI)	*P-value*
		0	1	≥ 2	≥ 1				
On ART*									
**No**	**3838**	3277(85.4)	298 (7.8)	263 (6.9)	561 (14.6)	1.00		1.00	
**Yes**	**7955**	7073(88.9)	442 (5.6)	440 (5.5)	882 (11.1)	0.76 (0.64–0.89)	< 0.001	0.68 (0.56–0.83)	< 0.001
On ART (VL^#^ <50 copies/mL)	6212	5545(89.3)	325 (5.2)	342 (5.5)	667 (10.7)	0.71 (0.60–0.84)	< 0.001	0.64 (0.51–0.79)	< 0.001
On ART (VL 50–999 copies/mL)	268	228(85.1)	23 (8.6)	17 (6.3)	40 (14.9)	1.02 (0.67–1.53)	0.937	0.69 (0.44–1.08)	0.631
On ART (VL ≥ 1000 copies/mL)	293	263(89.8)	16 (5.5)	14 (4.8)	30 (10.2)	0.67 (0.41–1.09)	0.110	0.65 (0.39–1.82)	0.490
On ART (VL unknown)	1182	1037(87.7)	78 (6.6)	67 (5.7)	145 (12.3)	0.82 (0.63–1.06)	0.131	0.89 (0.67–1.18)	0.425
**On ART ≥ 3 years** (VL < 50 copies/mL)	**1918**	1807(94.2)	72 (3.8)	39 (2.0)	111 (5.8)	0.35 (0.28–0.46)	< 0.001	0.29 (0.22–0.38)	< 0.001

**¶** OR: Odds ratio

* ART: Antiretroviral therapy

# VL: Viral load

Covariates in the adjusted model included: age, gender, ethnicity, education, marital status, occupation, route of transmission, year of diagnosis, subtype, and CD4 + counts at diagnosis

### Visualization of molecular linkage between baseline cases and newly diagnosed cases

**Fig. 5:**
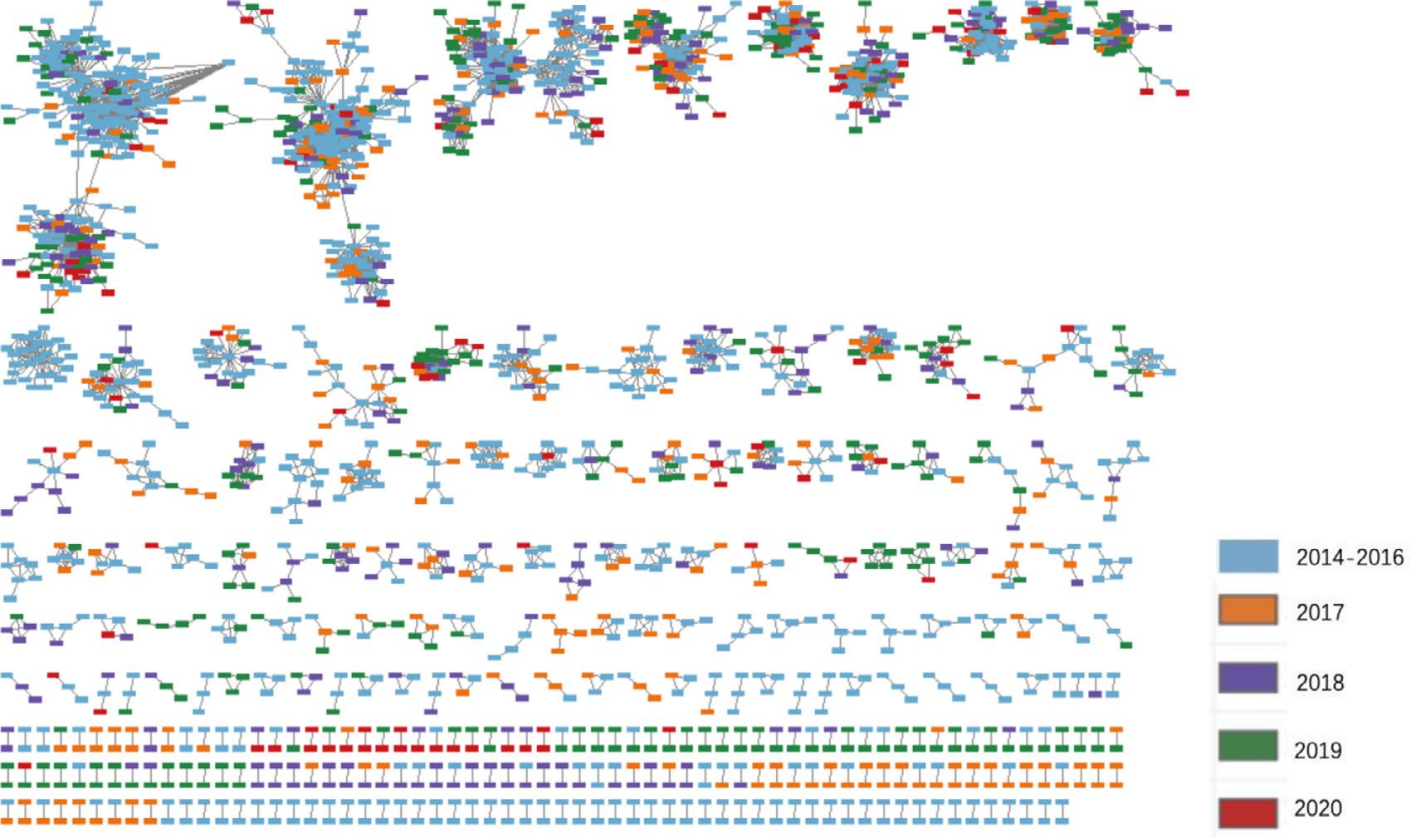
Visualization of the molecular linkages between baseline HIV/AIDS cases and newly diagnosed cases. Legend: The HIV molecular transmission network was constructed based on the genetic distance threshold of 0.0050 substitutions/site. The linkages between baseline cases diagnosed in 2014–2019 and newly diagnosed cases in 2017–2020 were illustrated. Blue denotes samples diagnosed in 2014–2016, orange denotes samples diagnosed in 2017, purple denotes samples diagnosed in 2018, green denotes samples diagnosed in 2019, red denotes samples diagnosed in 2020. 1879 cases out of 4270 total samples were included and displayed, with 2391 singletons omitted

Figure 5 visualizes the linkages between HIV baseline cases diagnosed in 2014–2019 and newly diagnosed cases in 2017–2020, respectively. The map only shows the nodes of linked cases while singletons were not displayed. 1879 cases out of 4270 total samples were included and displayed, with 2391 singletons omitted. It can be seen that HIV cases newly diagnosed in 2017, 2018 and 2019 had more linkages with corresponding baseline cases than those newly diagnosed in 2020.

### Sensitivity analysis

To support our finding, a sensitivity analysis was conducted based on different genetic distance thresholds (0.0025 and 0.0100 substitutions/site). The models included the same covariates as those in the previous GEE models. Additional file 1 shows the results of associations between ART status, HIV viral load level and HIV transmission under the two genetic distance thresholds. In general, cases on ART regardless of HIV viral load level had a significantly lower risk of HIV transmission. Additionally, cases on ART and a viral load level less than 50 copies/mL had a significantly lower HIV transmission risk. Those with sustained ART for at least three years and a viral load level less than 50 copies/mL possessed robust prediction capability in reducing the risk of HIV transmission.

## Discussion

Results of this longitudinal molecular network study indicate that patients on ART have a 32% reduction in HIV transmission compared with those not on ART at the population level. The effectiveness of treatment as a prevention has been shown by several observational prospective cohort studies among sero-discordant couples [5, 6, 25–27] and further at the population level by one randomized clinical trial HPTN 071 (PopART) applying universal HIV testing and ART in the real-world setting [10]. A previous study based on a longitudinal molecular study reported a 53.6% reduction in HIV transmission without considering any demographic and clinical characteristics [19]. Our results for ART prevention efficacy of HIV transmission with adjustment for demographic and clinical variables in the multivariate models may be more accurate. HIV can be well inhibited by ART [28] and the transmission risk is related to the high viral load in a patient [29]. Based on our stratification analysis of HIV viral loads, HIV transmission can be reduced by 36% if the viral load is less than 50 copies/mL. Further, we identified that cases with sustained ART of at least 3 years and with a viral load lower than 50 copies/mL can reduce transmission by 71%. The genetic distance threshold of 0.0050 refers to a 2–3 years evolution time of the HIV strain [11]. Hence, irrelevant genetic linkages can be removed in the molecular network when patients have received ART for more than 3 years. The linkage indicated the real effect of HIV transmission under the context of ART expansion in the communities. Our findings strongly support the WHO’s recommendation of “treat all” policy at the population level.

We found that patients who dropped out of ART had the same risk of HIV transmission as those not on ART. The dropout rate for patients who started ART is considerably high in China, especially in those with high CD4 + counts or those who started ART within the first year [30, 31]. HIV/AIDS cases who dropped out of ART have a higher risk of increased drug resistance and viral load rebound [32]. This could lead to more secondary HIV transmissions in the communities. This indicates that ART should be promoted in communities for HIV prevention, but its effect will not be seen until the viral load in a patients’ body falls below 50 copies/mL. Hence, adherence to ART is crucial in increasing the effectiveness of HIV treatment [33]. China has implemented an early treatment policy to all HIV/AIDS patients regardless of CD4 + counts since late 2016 [9]. To meet the request of UNAIDS’s three “95” targets, ensuring that everyone adheres to ART is currently the top priority in the context of scaling up ART. Strengthening PLHIV’s relationships with their spouse/partner and children, promoting collaborative provider-patient relationships, and enhancing peer support among PLHIV can also increase ART adherence [34]. Advanced techniques should be applied in future treatment management of PLHIV, such as providing preferred digital strategies to distribute ART medication messages, medication reminders, peer education, and involvement in online discussions [35]. HIV viral load monitoring for HIV/AIDS cases with higher CD4 + counts at diagnosis is also necessary for ensuring improved long-term treatment outcomes under the scaling up of ART in the communities [19].

Our study identified that the patients who were older, male, non-Han, retired, and with higher CD4 + counts had a higher risk of HIV transmission. The number of elderly HIV/AIDS cases accounts for a large part of the yearly routine newly diagnosed HIV/AIDS cases in Guangxi [36] and their mortality is higher than other age groups [31]. The basic baseline cases in 2014–2016 were current HIV/AIDS cases as of December 31, 2016 and were not limited to newly diagnosed cases. This cohort contained more younger cases. Therefore, the proportion of elderly cases in the total sample in 2014–2016 may be lower than those in newly diagnosed cases in 2017, 2018, 2019, and 2020. A community based study conducted in Guangxi also discovered that older men have a higher risk of HIV transmission than other age groups [37].The main reason might be that elderly prefer to have sexual encounters with low-cost sexual workers who already possess a high HIV prevalence [38, 39]. It is indicated that cases with a higher CD4 + count yield a higher risk, consistent with other studies [40]. The reason for this might be they are more recently diagnosed and not aware of their HIV infection status previously; they may not purposely take protected measures when having sexual encounters. Additionally, such cases could still consider themselves to be healthy and tend to drop out, even after initiating ART. This would make HIV suppression difficult. When HIV is not effectively suppressed, HIV transmission could occur easily, especially during unprotective sexual behaviors. We found that those infected via heterosexual intercourse had a higher HIV transmission risk. This might due to genetic differences between the HIV-1 transmitted/founder strains in different transmission modes [41]. HIV-1 CRF01_AE is less transmissible than other subtypes because it is associated with poorer clinical outcomes and faster CD4 T-cell decline, resulting in patients dying faster [42]. Only the baseline network containing cases diagnosed in 2014–2019 had fewer linkages with newly diagnosed HIV cases in 2020 compared with linkages that occurred between other baseline cases and newly diagnosed cases. This was consistent with the molecular mapping, linkages between newly diagnosed cases and baseline cases did not reduce dramatically until 2019. All of the findings showing associations between socio-demographic and clinical characteristics and HIV secondary transmission were consistent with the previous results achieved from a large-scale prospective cohort study conducted in Guangxi [37]. This indicates that HIV secondary transmission has been decreasing gradually under the expansion of ART in the communities.

Our study has some limitations. Firstly, we could not achieve HIV sequences for all newly diagnosed cases during 2014–2020 due to insufficient blood samples. This could result in underestimation of the linkages between baseline cases and newly diagnosed cases. Since some missing cases did not appear in the molecular network, the transmission between the linked cases seen in the network may not reflect the real world. Newly diagnosed cases might be infected by other cases not shown in the molecular network - their linkage may be because they have a similar virus under a certain genetic distance threshold, and overestimation of HIV transmission can occur according to this kind of linkage. Secondly, we used HIV-1 *pol* region for the study; there is a genomic evolution rate of 1% every 10 years in population level for *pol* region [11]. Under a current constant genetic distance threshold of 0.05% substitutions/site employed in molecular transmission network construction, a small part of HIV transmission caused by cases evolved faster or slower would be missed as determined in our study. Thirdly, the HIV transmission caused by cases in 2014–2016 decreased, but the new infections driven by newly diagnosed cases in 2017, 2018, 2019, 2020, respectively cannot be determined properly by our study since the observation time is not long enough yet. Finally, there might be some drug resistant mutations in the sequences, but a former study found that there is consistent identification of molecular linkages for certain sequences regardless of inclusion or exclusion of drug resistant mutations [23].

### Additional files

The online version contains Additional files available at XXXXXX.

### Electronic supplementary material

Below is the link to the electronic supplementary material.

Supplementary Material 1: Additional file 1. Sensitivity analysis of HIV transmission of molecular linkages between baseline HIV/AIDS cases during 2014–2019 and newly diagnosed cases during 2017–2020 in Qinzhou, Guangxi, respectively.

## Data Availability

The HIV sequences used in this study are not publicly available but can be obtained via reasonable request and approval from the Chinese Center for Disease Control and Prevention. Requests to access these datasets should be directed to YR, ruanyuhua92@chinaaids.cn.

## Declarations

### Transparency declarations

None to declare.

## References

[1] Cohen MS, Chen YQ, McCauley M, Gamble T, Hosseinipour MC, Kumarasamy N (2016). Antiretroviral therapy for the Prevention of HIV-1 transmission. N Engl J Med.

[2] Grinsztejn B, Hosseinipour MC, Ribaudo HJ, Swindells S, Eron J, Chen YQ (2014). Effects of early versus delayed initiation of antiretroviral treatment on clinical outcomes of HIV-1 Infection: results from the phase 3 HPTN 052 randomised controlled trial. Lancet Infect Dis.

[3] Cohen MS, Chen YQ, McCauley M, Gamble T, Hosseinipour MC, Kumarasamy N (2011). Prevention of HIV-1 Infection with early antiretroviral therapy. N Engl J Med.

[4] Tang Z, Lan G, Chen YQ, Zhu Q, Yang X, Shen Z (2015). HIV-1 treatment-as-Prevention: a cohort study analysis of serodiscordant couples in Rural Southwest China. Med (Baltim).

[5] Smith MK, Westreich D, Liu H, Zhu L, Wang L, He W (2015). Treatment to prevent HIV transmission in serodiscordant couples in Henan, China, 2006 to 2012. Clin Infect Dis off Publ Infect Dis Soc Am.

[6] Jia Z, Mao Y, Zhang F, Ruan Y, Ma Y, Li J (2013). Antiretroviral therapy to prevent HIV transmission in serodiscordant couples in China (2003–11): a national observational cohort study. The Lancet.

[7] UNAIDS., Fast-Track: ending the AIDS epidemic by 2030; 2014.

[8] Cai WP, Chen XJ, Li HQ (2016). Manual of the National Free Antiretroviral Treatment.

[9] Yang X, Wang Z, Wang X, Ma T, Xue H, He Y (2019). Behavioral intention to initiate antiretroviral therapy (ART) among Chinese HIV-Infected men who have sex with men having high CD4 count in the era of treatment for all. Am J Mens Health.

[10] Hayes RJ, Donnell D, Floyd S, Mandla N, Bwalya J, Sabapathy K (2019). Effect of Universal Testing and Treatment on HIV incidence - HPTN 071 (PopART). N Engl J Med.

[11] USCDC (2018). Detecting and responding to HIV transmission clusters - a guide for Health departments.

[12] Wertheim JO, Murrell B, Mehta SR, Forgione LA, Kosakovsky Pond SL, Smith DM (2018). Growth of HIV-1 molecular transmission clusters in New York City. J Infect Dis.

[13] Poon AFY, Gustafson R, Daly P, Zerr L, Demlow SE, Wong J (2016). Near real-time monitoring of HIV transmission hotspots from routine HIV genotyping: an implementation case study. Lancet HIV.

[14] Dennis AM, Volz E, Frost ASMSDW, Hossain M, Poon AFY, Rebeiro PF (2018). HIV-1 transmission clustering and Phylodynamics highlight the important role of Young men who have sex with men. AIDS Res Hum Retroviruses.

[15] Whiteside YO, Song R, Wertheim JO, Oster AM (2015). Molecular analysis allows inference into HIV transmission among young men who have sex with men in the United States. AIDS Lond Engl.

[16] Chen Y, Lan G, Feng Y, Ruan Y, Shen Z, McNeil EB et al. Inferring potential non-disclosed men who have sex with men among self-reported heterosexual men with HIV in Southwest China: A genetic network study. Li Y, editor. PLOS ONE. 2023;18(3):e0283031. 10.1371/journal.pone.0283031 PMC10065240 37000807

[17] Ragonnet-Cronin M, Hué S, Hodcroft EB, Tostevin A, Dunn D, Fawcett T (2018). Non-disclosed men who have sex with men in UK HIV transmission networks: phylogenetic analysis of surveillance data. Lancet HIV.

[18] Ragonnet-Cronin M, Hu YW, Morris SR, Sheng Z, Poortinga K, Wertheim JO (2019). HIV transmission networks among transgender women in Los Angeles County, CA, USA: a phylogenetic analysis of surveillance data. Lancet HIV.

[19] Kang R, Li J, Chen H, Tang Z, Pan SW, Luo L (2021). Using longitudinal genetic-network study to understand HIV treatment-as-prevention. AIDS.

[20] Liang KY, Zeger SL (1986). Longitudinal data analysis using generalized linear models. Biomttrika.

[21] Chen H, Luo L, Pan SW, Lan G, Zhu Q, Li J (2019). HIV Epidemiology and Prevention in Southwestern China: Trends from 1996–2017. Curr HIV Res.

[22] Xing H, Ruan Y, Hsi JH, Kan W, Liao L, Leng X et al. Reductions in virological failure and drug resistance in Chinese antiretroviral-treated patients due to lamivudine-based regimens, 2003-12. J Antimicrob Chemother. 2015;dkv078. 10.1093/jac/dkv078 25855758

[23] Wertheim JO, Kosakovsky Pond SL, Forgione LA, Mehta SR, Murrell B, Shah S et al. Social and Genetic Networks of HIV-1 Transmission in New York City. Bonhoeffer S, editor. PLOS Pathog. 2017;13(1):e1006000. 10.1371/journal.ppat.1006000 PMC5221827 28068413

[24] Penn state Eberly college of science. STAT 504. Analysis of Discrete Data.12.1 - Introduction to Generalized Estimating Equations. https://online.stat.psu.edu/stat504/lesson/12/12.1 Accessed 17 October 2023.

[25] Chen H, Yang X, Zhu Q, Wu X, Chen L, Lu H (2018). Treatment for HIV prevention study in southwestern areas of China. Infect Dis Model.

[26] Nanditha NGA, Dong X, Tafessu HM, Wang L, Lu M, Barrios R (2022). A province-wide HIV initiative to accelerate initiation of treatment-as-prevention and virologic suppression in British Columbia, Canada: a population-based cohort study. CMAJ Open.

[27] Herce ME, Hoffmann CJ, Fielding K, Topp SM, Hausler H, Chimoyi L (2020). Universal test-and-treat in Zambian and South African correctional facilities: a multisite prospective cohort study. Lancet HIV.

[28] Bavinton BR, Pinto AN, Phanuphak N, Grinsztejn B, Prestage GP, Zablotska-Manos IB (2018). Viral suppression and HIV transmission in serodiscordant male couples: an international, prospective, observational, cohort study. Lancet HIV.

[29] Eisinger RW, Dieffenbach CW, Fauci AS (2019). HIV viral load and transmissibility of HIV Infection: undetectable equals untransmittable. JAMA.

[30] Tang Z, Pan SW, Ruan Y, Liu X, Su J, Zhu Q (2017). Effects of high CD4 cell counts on death and attrition among HIV patients receiving antiretroviral treatment: an observational cohort study. Sci Rep.

[31] Zhu J, Yousuf MA, Yang W, Zhu Q, Shen Z, Lan G (2021). Mortality and attrition rates within the First Year of antiretroviral therapy initiation among people living with HIV in Guangxi, China: an Observational Cohort Study. BioMed Res Int.

[32] Lei L, Zhongbao Z, Lingjie L, Shujia L, Yanling M, Guohui W (2018). The drug resistance in HIV/AIDS patients who had stopped art in 2016. J Trop Med.

[33] Abuogi LL, Onono M, Odeny TA, Owuor K, Helova A, Hampanda K (2022). Effects of behavioural interventions on postpartum retention and adherence among women with HIV on lifelong ART: the results of a cluster randomized trial in Kenya (the MOTIVATE trial). J Int AIDS Soc.

[34] Mao Y, Qiao S, Li X, Zhao Q, Zhou Y, Shen Z, Depression (2019). Social Support, and adherence to antiretroviral therapy among people living with HIV in Guangxi, China: a longitudinal study. AIDS Educ Prev.

[35] Jiao K, Wang C, Liao M, Ma J, Kang D, Tang W (2022). A differentiated digital intervention to improve antiretroviral therapy adherence among men who have sex with men living with HIV in China: a randomized controlled trial. BMC Med.

[36] Ge X et al. Yang Wenmi,Zhu Qiuying,Wu Xiuling,Shen Zhiyong,Zhu Jinhui,. Epidemiological characteristics of HIV/AIDS in Guangxi Zhuang Autonomous Region,2010–2017. Chin J Epidemiol,2019;40(3):315–321. 10.3760/cma.j.issn.0254-6450.2019.03.011 30884610

[37] Chen H, Wu X, Chen L, Lu H, Tang Z, Shen Z (2019). Rapidly spreading human immunodeficiency Virus Epidemic among older males and Associated factors: a large-scale prospective cohort study in Rural Southwest China. Sex Transm Dis.

[38] Chen X, Qin C, Chen R, Huang Y, Xu Y, Tang Q (2021). Epidemiological profile and molecular genetic characterization of HIV-1 among female sex workers and elderly male clients in Guangxi, China. Emerg Microbes Infect.

[39] Chen Y, Shen Z, Morano JP, Khoshnood K, Wu Z, Lan G (2015). Bridging the epidemic: a Comprehensive Analysis of Prevalence and correlates of HIV, Hepatitis C, and Syphilis, and Infection among Female Sex Workers in Guangxi Province, China. PLoS ONE.

[40] Fabeni L, Alteri C, Berno G, Scutari R, Orchi N, De Carli G (2019). Characterisation of HIV-1 molecular transmission clusters among newly diagnosed individuals infected with non-B subtypes in Italy. Sex Transm Infect.

[41] James A, Dixit NM (2022). Transmitted HIV-1 is more virulent in heterosexual individuals than men-who-have-sex-with-men. PLoS Pathog.

[42] Gangcuangco LMA, Eustaquio PC (2023). The state of the HIV Epidemic in the Philippines: Progress and challenges in 2023. Trop Med Infect Dis.

