# Supplementary material for: HIV transmission and associated factors under the scale-up of HIV antiretroviral therapy: a population-based longitudinal molecular network study
Source: Virol J. 2023 Dec 4;20:289. doi: 10.1186/s12985-023-02246-1 (PMC10696835; doi:10.1186/s12985-023-02246-1)
Supplement: Supplementary file 1 — Supplementary Material 1: Additional file 1. Sensitivity analysis of HIV transmission of molecular linkages between baseline HIV/AIDS cases during 2014–2019 and newly diagnosed cases during 2017–2020 in Qinzhou, Guangxi, respectively. [file 12985_2023_2246_MOESM1_ESM.docx]

**Additional file 1. Sensitivity analysis of HIV transmission of** **molecular linkages between baseline HIV/AIDS cases during 2014-2019 and newly diagnosed cases during 2017-2020 in Qinzhou, Guangxi, respectively.**

| **Genetic**  **distance threshold** | **ART* status** | **Baseline cases** | **Linkages between newly diagnosed cases and baseline cases, n (%)** | | | **Adjusted OR^¶^ (95%CI)** | ***P-value*** |
| --- | --- | --- | --- | --- | --- | --- | --- |
|  |  |  | **1** | **≥2** | **≥1** |  |  |
| 0.25% | Total | 11793 | 313 (2.7) | 203 (1.7) | 516 (4.4) |  |  |
|  | On ART |  |  |  |  |  |  |
|  | No | 3838 | 125 (3.3) | 62 (1.6) | 187 (4.9) | 1.00 |  |
|  | Yes | 7955 | 188 (2.4) | 141 (1.8) | 329 (4.1) | 0.56 (0.42-0.74) | <0.001 |
|  | - On ART & VL^#^ <50 copies/mL | 6212 | 140 (2.3) | 96 (1.5) | 236 (3.8) | 0.47 (0.35-0.64) | <0.001 |
|  | - On ART ≥ 3 years &VL<50 copies/mL | 1918 | 4 (0.2) | 2 (0.1) | 6 (0.3) | 0.10 (0.04-0.23) | <0.001 |
| 1.00% | Total | 11793 | 1386 (11.8) | 2688 (22.7) | 4074 (34.5) |  |  |
|  | On ART |  |  |  |  |  |  |
|  | No | 3838 | 492 (12.8) | 971 (25.3) | 1463 (38.1) | 1.00 |  |
|  | Yes | 7955 | 894 (11.2) | 1717 (21.6) | 2611 (32.8) | 0.81 (0.71-0.93) | 0.002 |
|  | - On ART & VL<50 copies/mL | 6212 | 457 (7.4) | 849 (13.7) | 1306 (21.0) | 0.73 (0.63-0.85) | <0.001 |
|  | - On ART ≥ 3 years &VL<50 copies/mL | 1918 | 186 (9.7) | 257 (13.4) | 443 (23.1) | 0.48 (0.40-0.58) | <0.001 |

*ART: Antiretroviral therapy

# VL: Viral load

**¶** OR: Odds ratio

Covariates of the adjusted model included: age, gender, ethnicity, education, marital status, occupation, route of transmission, year of diagnosis, subtype, CD4+ counts at diagnosis.
